# Age-Related Patterns of Midfacial Fractures in a Hungarian Population: A Single-Center Retrospective Study

**DOI:** 10.3390/jcm14155396

**Published:** 2025-07-31

**Authors:** Enikő Orsi, Lilla Makszin, Zoltán Nyárády, Lajos Olasz, József Szalma

**Affiliations:** 1 Department of Oral and Maxillofacial Surgery, Faculty of Medicine, University of Pécs, 7624 Pécs, Hungary; lajos.olasz@pte.hu (L.O.); jozsef.szalma@pte.hu (J.S.); 2 Institute of Bioanalysis, Faculty of Medicine, University of Pécs, 7624 Pécs, Hungary; lilla.makszin@pte.hu; 3 Nyárfa Dent Kft., 9024 Győr, Hungary; nyarady.zoltan@gmail.com

**Keywords:** midfacial fracture, elderly patient, facial trauma, low-energy injury, fall-related injury, zygomatic fracture, edentulism, hospitalization, associated injuries

## Abstract

**Background:** Midfacial fractures are common outcomes of facial trauma. While younger individuals typically sustain these injuries through high-energy events like assaults and traffic or sports accidents, elderly patients increasingly present with fractures from low-energy mechanisms, primarily falls. **Purpose:** The aim of this study was to analyze age- and gender-specific patterns in midfacial fractures over a 10-year period, with emphasis on elderly individuals and low-energy trauma. **Methods:** A retrospective review was performed of proven midfacial fractures between 2013 and 2022 at the Department of Oral and Maxillofacial Surgery (University of Pécs, Hungary). The patients were stratified by age (<65 vs. ≥65 years) and gender. The variables included the injury mechanism, fracture localization, the dental status, hospitalization, and the presence of associated injuries. Bivariate analyses were performed, and the significance level was set to *p* < 0.05. **Results:** A total of 957 radiologically confirmed midfacial fracture cases were evaluated, of whom 344 (35.9%) were ≥65 years old. In the elderly group, females had a 19-fold higher risk for midfacial trauma than younger females (OR: 19.1, 95%CI: 9.30–39.21). In the older group, a fall was significantly the most frequent injury mechanism (OR: 14.5; 95%CI: 9.9–21.3), responsible for 89.5% of the cases, while hospitalization (OR: 0.36; 95%CI: 0.23–0.56) was less characteristic. Most of the fractures occurred in the zygomatic bone, in the zygomaticomaxillary complex, or in the anterior wall of the maxilla. Associated injuries in the elderly group included mostly lower limb injuries—particularly pertrochanteric femoral fractures in females—and upper limb injuries, with a slight male dominance. **Conclusions:** Low-energy falls are the primary cause of midfacial fractures in elderly patients, particularly in women. Tailored prevention and management strategies are essential for improving the outcomes in this growing demographic group.

## 1. Introduction

Midfacial fractures constitute a substantial portion of maxillofacial trauma and are traditionally linked to high-energy mechanisms such as interpersonal violence and road traffic accidents [1,2,3,4]. However, the increasing proportion of elderly individuals in the global population has led to a notable shift in the etiology and presentation of such injuries. In this demographic, low-energy trauma—particularly ground-level falls—has become the predominant cause [5,6,7].

Elderly patients present unique anatomical and physiological challenges in the context of facial trauma. Age-related osteopenia and osteoporosis, in conjunction with edentulism and diminished facial structural support, significantly increase the risk of a fracture from relatively minor impacts [8,9,10]. These changes not only alter fracture patterns, but also influence management strategies [11,12,13,14]. In many cases, conservative treatment is favored due to limited physiological reserves, comorbidities, and reduced functional and aesthetic expectations among older patients [6,15,16,17].

In addition to skeletal fragility, edentulism represents a key anatomical and functional risk factor in elderly patients who sustain midfacial trauma [8,10]. Tooth loss compromises the vertical dimension of occlusion and significantly alters maxillary and mandibular support, reducing the capacity of the masticatory system to absorb external forces. Consequently, even minor low-energy impacts may translate into the direct transmission of force to the midfacial bones, increasing the likelihood of a fracture.

Few studies have comprehensively addressed the intersection of the age, gender, and injury mechanism in midfacial trauma, particularly in Central and Eastern European populations [2,18]. Understanding these variables is critical for optimizing preventive strategies and tailoring therapeutic protocols to meet the specific needs of elderly patients [19,20].

The present study aimed to retrospectively analyze a 10-year cohort of patients with midfacial fractures in Hungary, at the University of Pécs, the Department of Oral and Maxillofacial Surgery. Emphasis was placed on identifying age- and gender-specific trends, with particular emphasis on the role of low-energy trauma and its implications for diagnoses, treatment planning, and resource allocation in geriatric care.

## 2. Materials and Methods

This retrospective cohort study was conducted at the Department of Oral and Maxillofacial Surgery, the University of Pécs, Hungary, and included patients diagnosed with midfacial fractures between January 2013 and December 2022. Patient data were retrieved from institutional electronic health records using ICD-10 diagnostic codes corresponding to midfacial fractures. Patients were included if they had radiologically (with CT) confirmed fractures involving the midfacial region (e.g., zygomatic complex, maxilla, frontal bone, orbital wall, Le Fort I–II–III, or naso-orbito-ethmoid (NOE) regionand when complete clinical documentation was available. Patients were excluded if they had incomplete medical records or when the fractures were limited to the mandible or nasal bone without midfacial involvement.

Patient data were retrospectively extracted from electronic medical records. All the data were fully anonymized in accordance with the General Data Protection Regulation (GDPR) prior to the analysis. The study variables included demographic characteristics (age, gender), the etiology of the injury (fall, traffic accident, assault, sports, other), fracture localization (i.e., the anatomical site of the fracture), and the hospitalization status (admitted to our maxillofacial institution, treated in external departments, or managed only on an outpatient basis). The analyzed variables further included the presence and location of associated injuries (e.g., head, upper/lower limb, trunk, orbital/nasal region) and the dental status of the elderly patient group. The dentition status was categorized as (i) patients with minor tooth loss and/or fixed dental prostheses (fewer than 3 teeth missing); (ii) patients with major tooth loss (more than 3 but fewer than 10 teeth missing); (iii) edentulous or partially edentulous patients with removable prostheses, and finally, (iv) completely edentulous patients without any prosthetic rehabilitation.

The patients then were stratified into two age groups: <65 years and ≥65 years, based on the World Health Organization (WHO)’s definition of elderly populations.

### 2.1. Ethical Approval

This study was approved by the Institutional Review Board of the University of Pécs (reference number: KK63973-1/2024). Due to the retrospective nature of the study, the requirement for informed consent was waived.

### 2.2. Statistical Analysis

Descriptive statistics were used to summarize the patient demographics and clinical characteristics. Categorical variables were analyzed with Pearson’s chi-square test, or Fisher’s exact test where appropriate (i.e., when the expected cell counts were below 5). MedCalc was used to compute univariate odds ratios [21]. A *p*-value < 0.05 was considered statistically significant. All the statistical analyses were performed using IBM SPSS Statistics, version 28.0 (IBM Corp., Armonk, NY, USA).

## 3. Results

### 3.1. Patient Demographics

**Figure 1:**
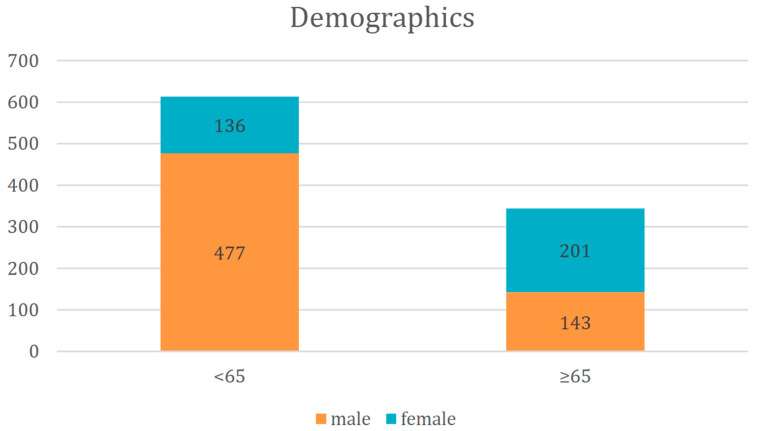
Distribution of patients by age and gender.

A total of 957 patients with CT-confirmed midfacial fractures were included in this study. Of these, 620 were male and 337 were female. Among the patients under the age of 65 (*n* = 613), the majority were male (*n* = 477), while females constituted the majority (*n* = 201) in the elderly group (≥65 years; *n* = 344) (Figure 1). The mean age of the study population was 50.73 years (SD ± 24.82). Male patients had a mean age of 48.66 years (SD ± 20.69), whereas female patients were significantly older, with a mean age of 66.1 years (SD ± 19.1). Stratified by age, the <65-year subgroup had a mean age of 39.7 years (SD ± 14.0), while the ≥65-year group had a mean age of 77.41 years (SD ± 7.6).

### 3.2. Dental Status in the Elderly Population

In the ≥65-year-old patient group (*n* = 344), the dental status of the patients was as follows: 28 patients had minor tooth loss and/or fixed dental prostheses (8.1%); 114 patients had major tooth loss (33.1%); 92 patients were edentulous or partially edentulous, but had removable prostheses (26.7%); and 110 patients were completely edentulous without any prosthetic rehabilitation (32.0%). Midfacial fractures showed a ~16-times higher occurrence (OR: 16.05, 95%CI: 10.32–24.98, *p* < 0.001) in patients with total or subtotal tooth loss than in patients with only minor (<3 teeth missing) tooth loss. A further analysis regarding the correlation of the dental status with specific fracture patterns was not performed in the primary analysis due to inconsistent documentation and subgroup heterogeneity.

### 3.3. Etiology of Midfacial Fractures

Falls were the most common cause of injury among elderly patients (≥65 years), accounting for 89.5% of cases, in contrast to 37.1% in the younger group (<65 years). In the <65 cohort, assaults (32.2%) and traffic accidents (22.1%), i.e., non-fall causes, were predominant when considering the etiology (Table 1). The chi-square test confirmed that this age-related difference in trauma mechanisms was statistically significant (*p* < 0.001).

### 3.4. Gender-Specific Injury Patterns

**Figure 2:**
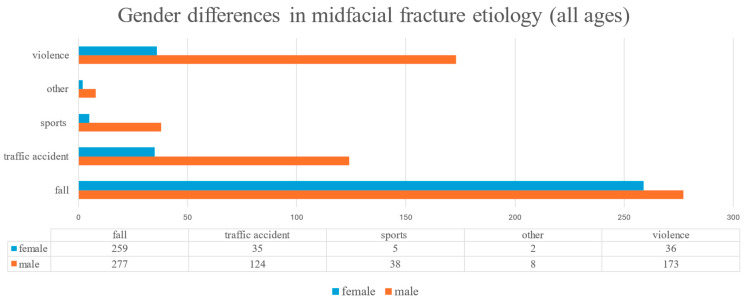
Gender-specific distribution of midfacial fracture etiology across all age groups.

**Figure 3:**
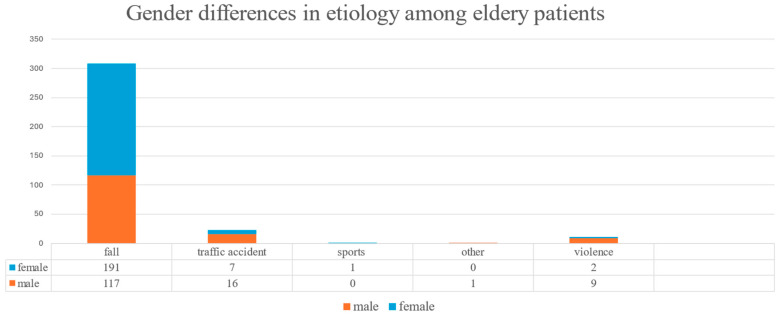
Gender differences in midfacial fracture etiology among elderly patients (≥65 years).

**Figure 4:**
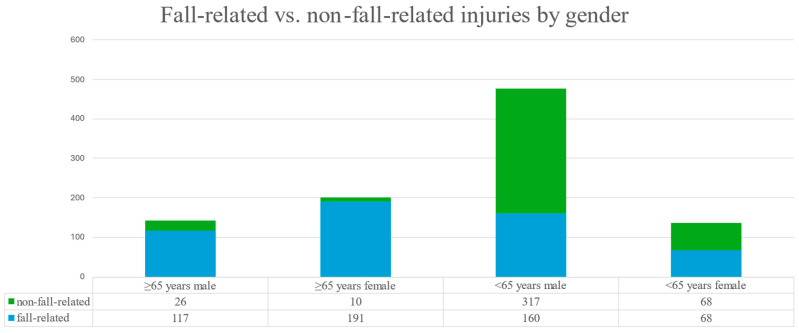
Distribution of fall-related vs. non-fall-related injuries by gender and age group.

Across all age groups, fall-related fractures were significantly more frequent in females (76.9%) than in males (44.7%). In the elderly subgroup, 95.0% of the fractures in females and 81.8% in males resulted from falls (Figure 2). Falls were more common in individuals aged 65 and older (89.5%) compared to those under 65 (37.1%) (Figure 3). Conversely, traffic accidents (6.7% vs. 22.2%), sports-related injuries (0.3% vs. 6.9%), and assault-related injuries (3.2% vs. 32.3%) were more frequent in the younger age group (Figure 4). The association among gender, age, and the fall-related etiology was statistically significant (*p* < 0.001).

An age- and sex-stratified analysis revealed significant differences in the fall-related midfacial fracture risk. Elderly men (≥65 years) had a nearly 10-fold higher chance for such injuries compared to younger men (OR = 9.92, 95%CI: 5.60–14.20, *p* < 0.001). This age-related increase was even more pronounced in women, where elderly females had a 19-fold higher risk compared to younger females (OR = 19.1, 95% CI: 9.30–39.21, *p* < 0.001) (Table 2).

In terms of gender differences, elderly females had a more than four-times higher chance of experiencing a fall-related fracture than their male counterparts (OR = 4.24, 95% CI: 1.98–9.12, *p* = 0.001). Among younger patients, females also showed a nearly twofold increased risk compared to younger males (OR = 1.98, 95% CI: 1.35–2.92, *p* < 0.001) (Table 2). These findings emphasize that the female sex and an advanced age act synergistically, placing elderly women at the highest overall risk for fall-related midfacial fractures.

### 3.5. Hospitalization Patterns

**Figure 5:**
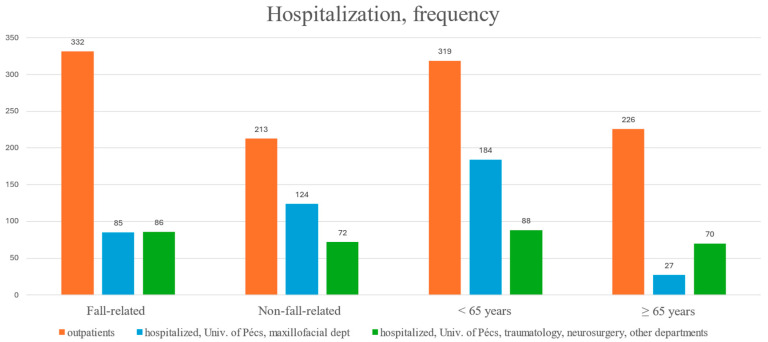
Hospitalization status by age and trauma etiology.

Patients with fall-related injuries were much more likely to be treated as outpatients, while non-fall-related injuries were more often managed in a hospital setting (Figure 5). Outpatient cases were ~6.6 times more likely to present fall-related injuries compared to hospitalized patients (Table 3, Figure 5). On average, one in five patients from the younger patient group (<65 years and one in ten patients from the elderly group (≥65 years) were hospitalized in our maxillofacial department (*p* < 0.001) (Table 3). However, when considering all kinds of hospitalizations (including those in other departments), 27.6% of elderly patients (≥65 years) and 20.4% of younger patients (<65 years) were treated as inpatients. Overall, elderly individuals were ~1.5 times more likely to be treated as inpatients; however, this occurred significantly less frequently in the maxillofacial department. The average length of hospitalization was 2.4 ± 1.3 days in our maxillofacial surgery department; it was 9.2 ± 3.7 days in other (traumatology, neurology, intensive care) departments.

### 3.6. Associated Injuries

**Figure 6:**
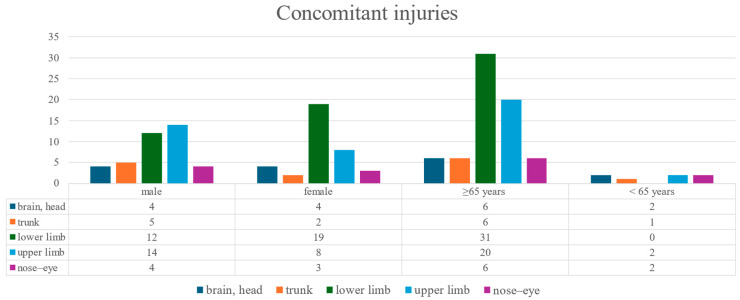
Distribution of concomitant injuries by anatomical region and patient group.

Among 957 patients, detailed data on the localization of concomitant injuries were available for 75 individuals. Notably, all 75 cases with documented concomitant injuries were hospitalized in external departments of the University of Pécs, primarily in the traumatology, otorhinolaryngology, or neurosurgery units (Figure 6). These records allowed for subgroup analyses based on both sex and age. The injury patterns differed notably by both sex and age. Elderly patients (≥65 years) most frequently sustained lower limb injuries (*n* = 31), which were almost exclusively absent in the younger group (<65 years). Upper limb injuries were also more common among the elderly *(n* = 20 vs. *n* = 2), while head/brain and trunk injuries were relatively evenly distributed. Females experienced more lower limb injuries (*n* = 19) than males (*n* = 12), whereas males showed a slightly higher frequency of upper limb and trunk trauma.

We recorded only the most severe injury in polytraumatized patients; therefore, for example, we did not see associated lower limb injuries in the younger age group.

### 3.7. Fracture Localization in Elderly Fall Patients

**Figure 7:**
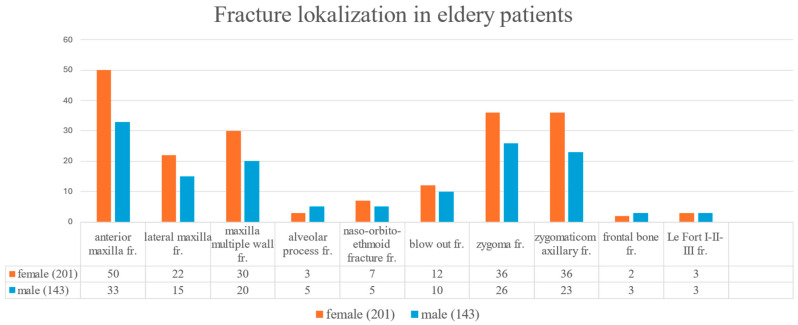
Anatomical localization of midfacial fractures in elderly patients with fall-related injuries.

Among elderly patients with fall-related injuries, the most frequently affected anatomical sites were the anterior maxillary wall (24.9%) the zygomaticomaxillary complex (17.9%), and the zygoma (17.9%). A statistically significant difference in fracture localization was found between elderly males and females (*p* = 0.047). Specifically, fractures involving the lateral maxillary wall (11.8% vs. 2.6%) and multiple maxillary walls (18.7% vs. 12.1%) were more frequent in women, while men had a higher proportion of zygomatic (24.1% vs. 17.6%) and zygomaticomaxillary (22.4% vs. 19.3%) fractures (Figure 7). These anatomical trends highlight the need for gender-sensitive diagnostic attention in geriatric trauma imaging.

## 4. Discussion

One of the central aims of this study was to identify demographic patterns in fall-related midfacial fractures. Table 4 was included to provide a structured overview of these patterns by age and gender. Importantly, it not only summarizes the findings of the present study, but also places them in context with relevant data from the international literature. This comparison helps to interpret the synergistic effects of an advanced age and the female sex as combined risk factors in midfacial trauma etiology. This retrospective analysis confirms that low-energy falls are the leading cause of midfacial fractures in elderly patients, especially among women. The disproportionately high incidence in females aged ≥65 may be attributed to several interrelated factors, including osteoporosis [1,4,6], edentulism [8,13], and a generally higher fall risk [13,20]. Demographic factors may also contribute in Hungary; women over the age of 65 outnumber men by nearly 1.5 to 1 [22], which likely influences the gender distribution observed in fall-related injuries. These findings align with previous studies emphasizing the vulnerability of the aging midface due to a reduced bone density and diminished structural resilience [8,13,14,20].

The fracture pattern in elderly patients differed markedly from that of younger individuals, with zygomatic and anterior maxillary wall fractures being most prevalent following falls [6,8,10,13,16]. In contrast, high-energy etiologies such as traffic accidents and interpersonal violence were more typical in the under-65 group [2,3,18]. The strong gender disparity in injury mechanisms—with women experiencing a higher rate of fall-related fractures—underscores the need for gender-sensitive prevention efforts.

The hospitalization rates were notably lower in elderly patients, and conservative management was more commonly employed in fall-related cases. This likely reflects both the low-impact nature of the trauma and a more cautious therapeutic approach, balancing surgical risks with expected functional benefits [6,15]. These trends echo findings in the maxillofacial trauma literature that advocate for individualized, age-adapted treatment plans in geriatric patients [3,5,23].

Our data also revealed that associated injuries—particularly to the lower extremities—are more common in elderly and female patients, suggesting the need for multidisciplinary trauma assessments and care [7,19]. The high prevalence of comorbidities and polypharmacy in this age group further reinforces the importance of integrating geriatric expertise into trauma management protocols [5,6,17,24]. Notably, international studies from Africa, India, Malaysia, and Poland have reported contrasting findings, with significantly fewer elderly patients and fall-related cases among midfacial fracture populations. This highlights the influence of differing demographic structures, healthcare access, and injury mechanisms across countries [9,14,18,19].

Table 4 summarizes the demographic characteristics of the studies referenced in the present analysis. Notably, in the reviewed literature as well as in our own data, fall-related midfacial fractures were typically associated with older age groups, further supporting the age-dependent nature of this trauma mechanism. Beyond surgical considerations, dental rehabilitation in elderly patients with midfacial trauma poses unique challenges. Age-related edentulism and a reduced bone quality often render even significant occlusal disturbances clinically less relevant, especially when the maxilla is involved [12,17]. Unlike mandibular trauma, maxillary fractures rarely result in complex prosthetic dilemmas due to the anatomical and functional characteristics of the upper arch [1,6,8,10]. However, a poor bone quality frequently contraindicates the use of dental implants [8], and the generally lower demand for advanced prosthetic care among elderly Hungarian patients compared to their Western European counterparts further reduces the clinical imperative for aggressive rehabilitation.

## 5. Limitations

This study has several limitations. First, its retrospective and single-center design may limit generalizability. Second, only the most severe associated injury was recorded in polytraumatized patients, which likely underestimates the total burden of trauma. Lastly, although the dentition status was documented in part, the data were incomplete and heterogeneous, which precluded a meaningful subgroup analysis regarding its influence on fracture patterns.

## 6. Conclusions

Midfacial fractures in elderly patients are predominantly the result of low-energy falls and often do not necessitate surgical intervention or hospitalization. The presence of comorbidities and elevated perioperative risks frequently supports a conservative treatment approach. Demographic factors—particularly a higher proportion of elderly women—appear to influence injury patterns. Recognizing these age- and gender-specific trauma profiles is essential for developing individualized care strategies and for avoiding overtreatment, particularly in cases where dental rehabilitation is unlikely to yield substantial functional or quality-of-life benefits due to anatomical limitations or socioeconomic constraints.

## Figures and Tables

**Table 1:** Distribution and odds ratios of injury mechanisms in patients aged <65 vs. ≥65 years.

Injury Mechanism	≥65 Years *n* (%)	<65 Years *n* (%)	Odds Ratio	95% Confidence Interval	*p*-Value
Fall	308 (89.5)	228 (37.2)	14.52	9.91–21.28	*p* < 0.001
Traffic accident	23 (6.7)	136 (22.2)	0.25	0.16–0.40	*p* < 0.001
Violence	11 (3.2)	198 (32.3)	0.07	0.04–0.13	*p* < 0.001
Sports	1 (0.3)	42 (6.9)	0.04	0.01–0.29	*p* < 0.001
Other	1 (0.3)	9 (1.5)	0.20	0.03–1.56	*p* = 0.11
Total	344 (35.9)	613 (64.1)			

**Table 2:** The correlation between gender, age, and fall-related injuries.

Comparison	Odds Ratio	95% Confidence Interval	*p*-Value
≥65 male vs. <65 male	9.92	5.60–14.20	<0.001
≥65 female vs. <65 female	19.1	9.30–39.21	<0.001
≥65 female vs. <65 male	4.24	1.98–9.12	=0.001
≥65 female vs. <65 male	1.98	1.35–2.92	<0.001

**Table 3:** Univariate odds ratios of the effect of injury mechanism and age on hospitalization.

		Injury Mechanism		Odds Ratio	95% Confidence Interval	*p*-Value
		Fall-related injury *n* (%)	Non-fall-related injury *n* (%)			
Outpatient		486	251			
**vs.**						
Hospitalized	Maxillofacial Dept.	20 (4.0)	125 (33.2)	0.08	0.05–0.14	<0.001
	Maxillofacial and other Departments	50 (9.3)	170 (40.4)	0.15	0.11–0.22	<0.001
		**Age**		**Odds Ratio**	**95% Confidence Interval**	***p*-Value**
		≥65 years *n* (%)	<65 years *n* (%)			
Outpatient		249	488			
**vs.**						
Hospitalized	Maxillofacial Dept.	27 (8.5)	118 (23.8)	0.36	0.23–0.56	<0.001
	Maxillofacial and other Departments	95 (27.6)	125 (20.4)	1.49	1.1–2.03	0.011

**Table 4:** Overview of age- and gender-specific patterns of fall-related midfacial fractures, as supported by findings in the present study and the relevant literature.

Oszlop1	Publication Year	Country	Study Focus	Main Etiological Factors (Top 3)	Number of Patients	Mean age (years)	Gender
Meisgeier et al. [11]	2024	Germany	Midfacial fractures; DRG database analysis	traffic accident, fall, violence,	374,147	44	72.94% M
Bettschen et al. [5]	2024	Switzerland	Elderly patients on anticoagulants with facial trauma	fall, traffic accident, violence	188	81	55.3% M
Giacomin et al. [8]	2017	Brazil	Elderly facial trauma, 10– year review	traffic accident, violence, fall	1385	NR	57.9% M
Atisha et al. [1]	2016	USA	Facial fractures in aging population	fall, traffic accident, assault	2023	39	61% M
Chávez Serna & Iñigo-Arroyo [12]	2021	Mexico	Facial fracture management in elderly, clinical experience	fall, traffic accident, assault	74	76.2	61% M
Yovev et al. [6]	2021	Germany	Surgical management of facial fractures in geriatric patients	fall, traffic accident, violence	300	78.8	39.3% M
Liu et al. [7]	2019	USA	Facial fractures in elderly due to falls	NR	139	75.7	49.6% M
Stay & Maschka [13]	2003	UK	Falls in elderly with facial injuries	fall, slip/trip, unclassified trauma	53	71.9	49% M
Béogo et al. [14]	2019	Burkina Faso	Facial fractures in the elderly	traffic accident, violence, animal attack	2400	NR	79.6% M
Kokko et al. [15]	2022	Finland	Associated injuries in elderly with facial trauma	violence, fall, traffic accident	2682	47.4	71.8% M
Agarwal et al. [9]	2025	India	Institutional experience in geriatric facial trauma	fall, traffic accident, assault	37	65	83.8% M
Matteo et al. [2]	2020	Europe (multicenter)	Maxillofacial trauma in elderly	NR	1334	79.3	44.9% M
Carvalho Filho et al. [10]	2015	Brazil	Oral/maxillofacial trauma in elders, hospital data	traffic accident, domestic accident, fall	47	72.4	44.79% M
Royan et al. [19]	2008	Malaysia	Facial fractures in elderly, developing country	traffic accident, assault, fall	1862	NR	81.18% M
Ogura et al. [3]	2016	Japan	Comparison of elderly vs. young in facial trauma	fall, assault, traffic accident	40	70.9	42% M
Kloss et al. [4]	2010	USA	Complications in midfacial fractures (surgical vs. conservative)	sports, traffic accident, violence	740	40.9	73% M

## Data Availability

The raw data supporting the conclusions of this article will be made available by the authors on request.
